# After 10 years, how do changes in asset ownership affect the *Indicador Econômico Nacional*?

**DOI:** 10.1590/S1518-8787.2017051006517

**Published:** 2017-02-23

**Authors:** Fernanda Ewerling, Aluísio J D Barros

**Affiliations:** I International Center for Equity in Health. Programa de Pós-Graduação em Epidemiologia. Universidade Federal de Pelotas. Pelotas, RS, Brasil

**Keywords:** Economic Indices, Social Conditions, Social Values, Socioeconomic Analysis

## Abstract

**OBJECTIVE:**

Our main objective was to analyse how the evolution of household assets ownership affected the *Indicador Econômico Nacional* (IEN – National Wealth index) and to point out the most stable assets and which lost importance more quickly.

**METHODS:**

We analysed the trend of the ownership of each IEN variable and the distribution of the households’ scores. We calculated the correlation coefficients of each variable separately with the IEN score and the household income. We also evaluated how the changes of the score distribution over time affected the validity of the published reference cut-points. We used data from consortium surveys conducted every two years from 2002 to 2014 in the city of Pelotas, Brazil.

**RESULTS:**

An increase in the educational level of household heads and in the ownership of all IEN assets, except radio and telephone, was observed in the study period. In general, the correlation of the assets with the IEN scores decreased over time. There was an increase in the score, with a consequent increase in the quintiles cut-points, but the distance between these cut-points had no significant variation. Thus, the reference cut-points for Pelotas, quickly became outdated.

**CONCLUSIONS:**

Some assets showed greatly reduction on its importance for the indicator, and the reference cut-points became obsolete very quickly. It is essential for a standardized wealth (or asset) index with research purposes to be updated frequently, especially the cut-points of reference distribution.

## RESUMO

**OBJETIVO:**

Analisar como a evolução temporal da posse de bens domésticos afetou o Indicador Econômico Nacional e como essas mudanças afetaram o poder discriminatório do indicador.

**MÉTODOS:**

Analisou-se a evolução temporal da posse de cada uma das variáveis do Indicador Econômico Nacional, bem como da distribuição do escore dos domicílios. Utilizamos dados de inquéritos populacionais realizados bienalmente no município de Pelotas, RS, de 2002 a 2014. Foi calculado o coeficiente de correlação de cada variável isoladamente com o escore do Indicador Econômico Nacional e com a renda familiar. Avaliamos também como a variação da distribuição do escore ao longo do tempo afetou a validade da utilização dos pontos de corte de referência publicados.

**RESULTADOS:**

Houve aumento da escolaridade dos chefes das famílias e da posse de todos os bens, exceto rádio e linha telefônica no período. A correlação dos bens com o Indicador Econômico Nacional reduziu com o tempo. O escore aumentou, com consequente incremento nos pontos de corte dos quintis, mas a distância entre os pontos não teve variação importante. Assim, os pontos de corte de referência publicados para Pelotas rapidamente ficaram desatualizados.

**CONCLUSÕES:**

Alguns bens perderam a capacidade discriminatória e os pontos de corte ficaram obsoletos rapidamente. É essencial um indicador de bens padronizado para uso em pesquisa, que seja atualizado com frequência, em especial os pontos de corte da distribuição de referência.

## INTRODUCTION

Social epidemiology sustains that individuals’ health directly connects to their life conditions^1^. Its consolidation led epidemiological studies to include socioeconomic determinants investigation in their analyses^2^.

Individuals can be classified in several ways according to their socioeconomic status. Income and consumption indicators require long questionnaires for their proper determination. As their measurement is not the focus of health surveys, their use in these surveys is generally not possible^4^. Thus, researchers might choose carefully the most appropriate method for every specific situation, considering their strengths and limitations^3^.

A proxy for household wealth based on household assets and household characteristics (such as number of bedrooms) was proposed as an alternative^5^. The called wealth index (or asset index) is a practical way to classify the households according to their socioeconomic status. It was initially used in the Demographic and Health Survey (DHS), which lacks information on income and consumption. Instead of the current income, this indicator represents the permanent consumption capacity of the family^a^. Its main advantage is to rely on a limited set of variables that are easily collected in health surveys, even with low educated populations. It is also a more stable socioeconomic measure than current income, which is subject to significant fluctuations when recorded in a relatively short reference period^b^.

The *Indicador Econômico Nacional* (IEN – National Wealth index) was developed using the same approach of the DHS Wealth Index and is widely used in epidemiological research in Brazil^6-9^. Its use enables to calculate scores for households from information on the ownership of a set of assets, household characteristics and the household head’s education^2^. The *Critério Brasil*, developed by the *Associação Brasileira de Empresas e Pesquisas* (ABEP), also present a similar proposal. The IEN is advantageous because it is based on a national coverage sample and because it provides the reference distribution of scores for Brazilian capitals, states and large regions, as well as the national distribution. From these data, we can compare the study sample (e.g. sample of health care users of the Family Health Strategy) with the more suitable reference distribution^c^.

The wealth indices also have important limitations. Its result is a relative measure, which provides a ranking of the individuals, but does not allow the comparison between different populations. This is caused by the fact that an individual from the poorest quintile of a high-income country may be richer than one from the richest quintile of a middle or low-income country^4^. The reference distributions proposed with the IEN was an attempt to minimize this problem. The use of asset ownership information in the indicator composition, while making the classification more practical, may be a problem in a society experiencing rapid income and technology improvements since expensive assets soon become popular. Thus, periodic updates are required to avoid these indicators from losing discriminatory power.

In the last decades, Brazil experienced a systematic decrease in income inequality, with especially growth of the poorest families’ income and credit expansion^d^. Data from the “*Pesquisa de Orçamento Familiar*” budget survey showed a popularization of durable goods. That is, the poorer families also achieved access to these goods^10^. The country also experienced rapid expansion of access to mobile telephony, and the service became almost universal. The richest strata changed from fixed to mobile telephones^11^.

As the IEN was created from the 2000 Census data, the relative weights of the assets that compose the indicator probably have changed due to the country’s advances in that period. This study aimed to analyze the trends of the IEN assets ownership and describe how these changes affected the discriminatory power of the indicator. This will enable to identify the best types of assets or household characteristics to include in these indicators, favoring those that are more stable and avoiding those that lose importance more quickly.

## METHODS

This study is part of a research consortium conducted in 2014 in the urban area of Pelotas^12^, a medium-sized Brazilian municipality in the southern region, which comprehended several research topics. We also used data from 2002 to 2012 consortium surveys conducted in the same city. IEN was developed in 2005, so the 2002 and 2004 surveys lack information on the ownership of some IEN assets. Therefore, some analyses excluded those years. The target population of all consortia prior to 2014 was adults (≥ 20 years) living in the urban area of Pelotas, except for 2012, which also included adolescents. The older adults (> 60 years) health was the focus in 2014. Therefore, the logistics was different from the previous years for the data collection in the households with no older adults.

In all surveys, the sampling process happened in two stages: systematic selection of the census tracts according to the average household head’s income, with probability proportional to the tract’s size, followed by systematic household selection^13^. Due to the lack of income data in 2012, the average education of the household head was used to order the sectors to procced the sampling.

In 2014, a sample size calculation evaluated the proportion of households that owned each IEN component asset. The refrigerator obtained the largest sample size, with a 99.0% expected prevalence, using alpha error of 5.0% and power of 80.0%. We used a confidence limit of 0.5 percentage points. According to calculations made in the OpenEpi^e^ program, 1,519 households were necessary, of which 506 with and 1,013 without older adult residents.

As the 2014 consortium included only households with older adults, we initially applied a short questionnaire to collect basic information on the household residents (family composition questionnaire). One in every two households without older adults was selected to answer a questionnaire about the ownership of some household assets and household characteristics. We interviewed 1,937 domiciles (897 with and 1,040 with no older adults). We applied a weight to each household, equal to the inverse of the sampling probability: 1 was the weight for households with older adult residents; and 1/(*a/b*) for the other households, where *a* is the number of households with no older adults that were selected for the asset ownership questionnaire and *b* is the total number of households with no older adults among those selected in the tract.

A trained team conducted the interviews. In 2014, the interviewers and the field supervisors applied the questionnaire of asset ownership to households with no older adult residents, when these households were identified by the application of the family composition questionnaire. We considered losses and denials the interviews unaccomplished after at least three attempts on different days and times. A field supervisor performed the last attempts. To control data quality, inconsistencies were checked on a weekly basis and a reduced questionnaire with key questions was applied in a new supervisors’ visit to 10.0% of randomly selected interviewees.

The IEN component variables were: household head’s education (in years of study: 0-3/4-7/8-10/11 or more, incomplete higher/complete education); number of bedrooms; number of bathrooms (with shower and toilet); number of television sets (0/1/2/3+); number of vehicles (0/1/2+); ownership (yes/no) of assets: radio, refrigerator, DVD or video tape (VCR), freezer/duplex refrigerator; washing machine (a cheaper option of washing machine with less functions, called “*tanquinho*”, was excluded), microwave, telephone line, computer and air conditioner. We also analyzed vacuum cleaner, clothes dryer, dishwasher, portable computer, motorcycle, and internet (broadband or dial-up), pay television (yes/no) and housekeepers.

We assessed the time trend of the ownership of each IEN component variables, as well as the household scores using descriptive statistics and graphical analyses. The correlation between each IEN component and (1) the total household scores; and (2) the household income was calculated. The Research Ethics Committee of the *Faculdade de Medicina da Universidade Federal de Pelotas* approved this study and the mentioned surveys (Protocol 472.357/2013). All interviewees signed the informed consent form.

## RESULTS

Table 1 shows the increase in the level of the household heads’ education from 2002 to 2014. In 2002, 29.4% of the household heads had at least complete high school; in 2014, it increased to 42.2%. The number of bathrooms and bedrooms remained relatively constant. The ownership of radios decreased, while the televisions sets increased. The proportion of households with at least one car had no difference between 2002 and 2008, but increased since 2010. The ownership of refrigerator, freezer, washing machine, microwave, computer, air conditioner and vacuum cleaner increased in the period. In 2014, computer was disaggregated in desktops and portable computers. We found that notebooks/netbooks were already more prevalent than desktops (49.0% and 41.0%, respectively). DVD (or VCR) ownership increased from 2002 to 2012, but declined in 2014. Fixed telephone lines and housekeepers were diminishing. In 2014, 5.7% of the households had dishwashers, 23.8% had clothes dryers, and 19.6% had motorcycles. In addition, 60.4% and 49.3% of them had internet access and pay television in 2014, respectively.

**Table 1:** Time trends of the household head’s education, assets ownership, access to services and characteristics of the households. Pelotas, RS, Southern Brazil, 2002 to 2014.

Variable	2002	2004	2006	2008	2010	2012	2014^a^
						
	n = 1,542	n = 1,537	n = 1,506	n = 1,448	n = 1,393	n = 1,552	n = 1,929
						
	%	%	%	%	%	%	%
Household head’s education (years)							
0-3	19.1	16.8	18.7	15.1	17.4	13.1	13.5
4-7	36.1	35.0	30.9	28.3	28.7	27.4	28.2
8-10	15.4	15.9	17.8	16.5	16.0	16.5	16.2
≥ 11, incomplete higher education	17.5	21.7	21.6	24.3	25.8	26.5	27.4
Complete higher education	11.9	10.7	11.0	15.8	12.2	16.5	14.8
Ownership of assets (yes/no)							
Refrigerator	95.1	94.6	95.5	94.8	97.0	98.9	98.7
Color Television	93.9	92.9	96.5	97.9	98.4	98.4	98.9
Radio	93.4	93.1	94.7	92.9	87.9	84.2	80.1
DVD^b^	40.9	39.4	55.1	73.8	79.1	80.5	73.9
Washing machine	64.5	64.1	65.5	66.5	72.7	72.8	83.7
Freezer	33.8	34.5	36.9	41.1	45.8	53.8	55.6
Telephone line	-	73.5	67.5	58.7	54.2	48.5	38.3
Microwave	-	-	28.6	40.0	48.8	64.6	69.7
Computer	-	-	25.2	38.8	51.1	62.7	66.5
Vehicle	40.0	41.2	39.6	41.5	45.3	51.6	55.0
Vacuum cleaner^c^	33.3	31.7	37.1	37.7	39.5	46.4	47.4
Housekeeper^c^	9.4	8.1	8.8	9.7	7.3	7.9	6.2
Air conditioner	-	-	9.0	22.5	25.0	23.3	32.5
Dish washer^c^	-	-	-	-	-	-	5.7
Clothes dryer^c^	-	-	-	-	-	-	23.8
Motorcycle^c^	-	-	-	-	-	-	19.6
Access to services (yes/no)							
Internet^c^	-	-	-	-	-	-	60.4
Pay television^c^	-	-	-	-	-	-	49.3
Characteristics of the household	mean	mean	mean	mean	mean	mean	mean
Number of bedrooms	-	-	1.9	1.8	1.8	1.9	1.9
Number of bathrooms	1.3	1.4	1.4	1.4	1.4	1.5	1.4

^a^ We calculated the means and proportions of 2014 considering the weights of the observations.

^b^ DVD = video tape (VCR) or DVD.

^c^ Assets and services that do not compose the National Wealth Index.

The household characteristics, as well as the household head’s education and the number of televisions and vehicles had a stable correlation with the IEN in the period (Table 2). Refrigerator, freezer and DVD were the variables with the greatest decrease in this correlation. Number of bedrooms, radio and air conditioner showed positive variation. Radio did not present a trend of decreased, only a lower correlation in 2006 compared to the following years. Internet, presented the highest correlation with the indicator, but was first collected in 2014.

**Table 2:** Pearson’s correlation coefficients between the continuous score of the National Wealth Index and the ownership of household assets/characteristics. Pelotas, RS, Southern Brazil, 2006 to 2014.

Variable	Correlation coefficient					

	2006	2008	2010	2012	2014^a^	Var%^b^
Household head’s education^c^	0.63	0.63	0.60	0.59	0.59	-6.3
Number of bedrooms (1/2/3/4 or more)	0.36	0.39	0.40	0.44	0.43	19.4
Number of bathrooms (0/1/2/3 or more)	0.66	0.65	0.62	0.62	0.59	-10.6
Number of televisions (0/1/2/3 or more)	0.72	0.69	0.70	0.65	0.65	-9.7
Number of vehicles (0/1/2 or more)	0.66	0.67	0.66	0.70	0.62	-6.1
Ownership of assets (yes/no)						
Refrigerator	0.20	0.27	0.13	0.13	0.15	-25.0
Radio	0.17	0.22	0.22	0.22	0.21	23.5
DVD^d^	0.64	0.56	0.53	0.43	0.45	-29.7
Washing machine	0.60	0.62	0.59	0.58	0.50	-16.7
Freezer	0.56	0.53	0.50	0.48	0.39	-30.4
Telephone line	0.54	057	0.54	0.53	0.43	-20.4
Microwave	0.65	0.69	0.66	0.59	0.56	-13.8
Computer	0.72	0.73	0.72	0.67	0.65	-9.7
Vacuum cleaner^e^	0.57	0.58	0.58	0.55	0.50	-12.3
Housekeeper^e^	0.37	0.39	0.29	0.36	0.30	-18.9
Air conditioner	0.48	0.48	0.50	0.61	0.59	22.9
Dishwasher^e^	-	-	-	-	0.30	-
Clothes dryer^e^	-	-	-	-	0.41	-
Motorcycle^e^	-	-	-	-	0.10	-
Access to services (yes/no)						
Internet^e^	-	-	-	-	0.62	-
Pay television^e^	-	-	-	-	0.48	-

^a^ We calculated the coefficients for 2014 considering the sample weights.

^b^ Percentage variation in the correlation coefficients between 2014 and 2006.

^c^ Variable categorized according to years of study: 0 (0-3 years); 1 (4-7 years); 2 (8-10 years); 3 (11 years or more, incomplete higher education); 4 (complete higher education).

^d^ DVD = video tape (VCR) or DVD.

^e^ Assets and services that do not compose the National Wealth Index.

The household income and the IEN continuous score correlation evaluation showed moderate value in all years, ranging from 0.43 in 2010 to 0.62 in 2008 (results not shown).

**Figure 1:**
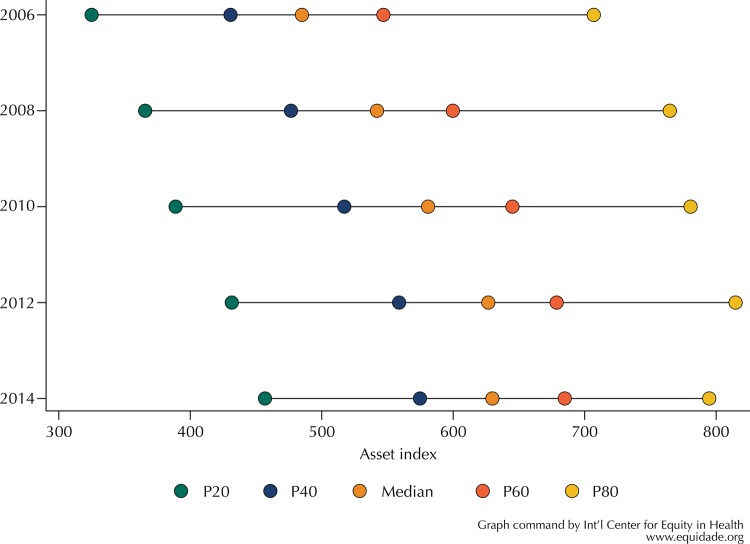
Cut-off points of the quintiles of the National Economic Indicator and its median. Pelotas, RS, Southern Brazil, 2006 to 2014.

The cutoffs for IEN quintiles had a consistent increase from 2006 to 2014. Figure 1 shows that the minimum score in 2006 and 2008 was 20 points, and it increased to 125 points in 2014. The distances between the cut-off points had no substantial variation, except for the 5th quintile cutoffs in 2014, which decreased compared with 2012.

**Figure 2:**
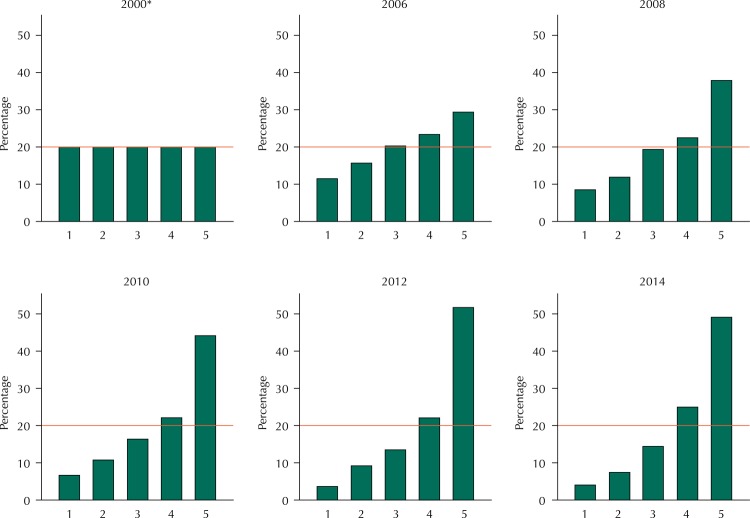
Distribution of the National Wealth Index for the consortia samples using the Pelotas reference quintiles calculated from the 2000 Demographic Census data. Pelotas, RS, Southern Brazil, 2006 to 2014.

The cut-off points of the reference quintiles calculated for Pelotas with the 2000 demographic census sample data showed a tendency of diminishing the size of the lowest reference quintiles and increasing the highest quintiles in the IEN distribution for the surveys from 2006 to 2014 (Figure 2).

* The 2000 chart presents hypothetical distribution data for this year.

**Figure 3:**
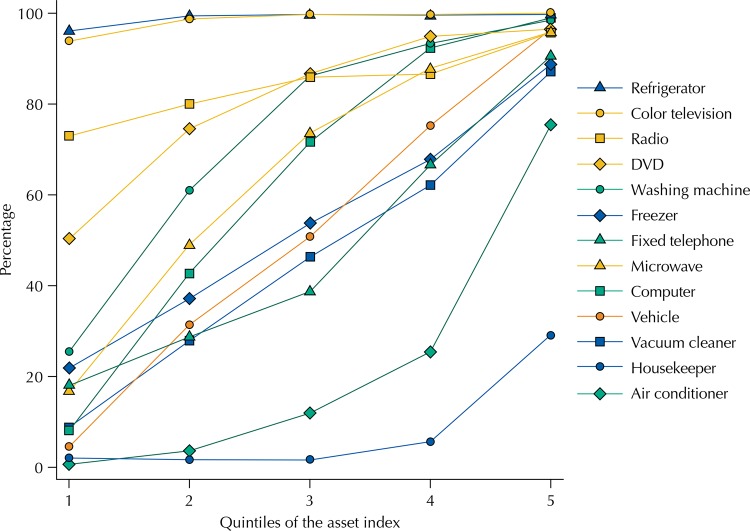
Percentage of households that own each good per quintiles of the National Wealth Index. Pelotas, RS, Southern Brazil, 2012.

All assets tended to increase their ownership in agreement with the IEN quintiles using the 2012 data, but with different trends (Figure 3). Assets as refrigerator, television, radio and DVD were common in the poorest group and virtually universal among the richest 20.0%. Other assets had approximately linear growth, such as vehicle, microwave, vacuum cleaner and computer. Air conditioner and housekeeper appeared in the richest quintile only.

## DISCUSSION

Selecting assets for a wealth indicator is a difficult task. Results showed that some variables are clearly markers of rich households, such as the air conditioner and housekeeper. Many assets are linear with quintiles, such as vehicle and freezer. Other assets are almost universal, such as refrigerator and television. Representatives of all these groups are important to maintain the discrimination capacity of the indicator, but including assets with very similar behavior represent no substantial discriminatory gain.

The prospect of replacement of these assets is also an important aspect. Basic appliances (e.g. television, refrigerator and microwave) are not likely to be substituted soon. Although increasingly popular, these assets should continue to be used. However, equipment such as radios and DVDs, with the growing supply of music, television and film programming thru pay television and the internet, are uncertain. These types of assets should be avoided. Common assets, such as television, can have a high correlation with wealth if considered the number of them in the household. It is improbable for stove or washing machine, but feasible for television, air conditioning, computer, etc.

Education and number of bedrooms and bathrooms have high correlation with the wealth score and should always be part of the items. However, characteristics of the household that depend on the public authority (such as paving) instead of exclusively on the households’ purchasing power are inadvisable indicators (although we have not presented data on this).

Household heads’ education and ownership of assets in all income quintiles over the years increased consistently, corroborating the literature^10^. Ownership of assets rise is probably due to increased income, easier and cheaper credit access^f^. The cost reduction of these assets due to competition, technological advance or tax incentive might have also influence the increase of the ownership^14^ of some assets^f^. Therefore, an increase in household scores, as Figure 1 shows, was expected. However, the distances between the quintiles did not change substantially in the period, suggesting a generalized increase in the population score of assets, and maintaining the inequality between the groups. The correlation of the assets with IEN fell along the years, except for the number of bedrooms, radio and air conditioner. The radio correlation is stable because the variation increase between 2006 and 2014 is not a tendency of the period, but rather a point variation, since 2006 presented a lower correlation, which rose in 2008 and remained constant.

Moderate correlation occurred between the IEN score and the household income. The original IEN^2^ article also presented this outcome, with a 0.40 Pearson’s correlation coefficient. Studies show that assets indicators are generally bad proxy for consumption expenditure or current income^15^, they are in fact household wealth indicators.

Like all other socioeconomic indicators, wealth indicators based on ownership of assets have limitations. The way in which these indicators are constructed, using the ownership of durable assets in their composition, can also entail the need for periodic updates and evaluations, such as the one carried out in this study, to assess their discriminatory power. Moreover, the comparison of different populations is not possible because wealth indicators are relative measures. The reference distributions for the IEN tried to solve this problem^2^.

The reference cut-off points become unstable over time despite the interest of comparing the study sample to a reference distribution (Figure 2). In the 2006 consortium, we already observed a distant distribution from the reference one. Updating the cut-off points within one to two years would be necessary so that the reference distribution remained valid. Thus, the effect of increasing assets ownership over time would be excluded, enabling to compare the sample distribution with the reference calculated for the same period. The demographic census occurs every 10 years, so it would be necessary to use another data source to calculate reference cut-off points. The National Household Sample Survey (PNAD), an annual survey conducted by the Brazilian Institute of Geography and Statistics is a possible alternative.

Using data from the Pelotas consortia was of great advantage for this study, as it is a set of surveys applied in the same place every two years with similar methodology. In addition, having data for almost all variables since 2002, two years after the demographic census, which was the IEN database, offers us important information to understand how the changes in the period affected the score.

We developed the analyses of Figure 3 from the 2012 consortium data. In the most recent survey (2014), the data from households with no the older adult residents were from a sub-sample. Households with older adults are different from those with no older adult residents: on average, households with no older adults have higher IEN score (29.2 95%CI 11.6–46.7 difference). In addition, due to the different sampling and the high number of losses, especially in the sectors of higher socioeconomic level, these data are more subject to bias than those of 2012. The analyses of 2014 were weighted, but the use of weights might not have been effective in eliminating all possible biases.

Epidemiological research and inequality studies normally use asset (or wealth) indices considering their easy, fast and stable classification of the households according to their socioeconomic situation^2-4,16,17^. Although widely used, the selection of the component variables of these indicators lack of a “best practice manual” to improve their discrimination capacity and their stability over time. In general, these variables are chosen arbitrarily^18^.

This study shows that the best assets are those that can discriminate households and have high correlation with the indices (or with the household income), with no substantial variation in this correlation over time. We also advise against including items with similar distribution among the wealth subgroups.

## Footnotes

a Ferguson B, Gakidou E, Murray C. Estimating permanent income using indicator variables.Geneva: World Health Organization; 2003 [cited 2015 Jun 22]. Available from: http://www.who.int/healthinfo/paper44.pdf

b Liverpool-Tasie LS, Winter-Nelson A. Asset *versus* consumption poverty and poverty dynamics in the presence of multiple equilibria in rural Ethiopia. Washington (DC): International Food Policy Research Institute; 2010 [cited 2015 Jun 22]. Available from: https://www.ifpri.org/publication/asset-versus-consumption-poverty-and-poverty-dynamics-presence-multiple-equilibria-rural

c Associação Brasileira de Empresas de Pesquisa (ABEP). Critério Brasil. São Paulo (SP); 2015 [cited 2015 Jun 22]. Available from: http://www.abep.org/criterio-brasil

d Fernandes MR. Estrutura e determinantes do consumo de bens duráveis no Brasil [dissertation]. São Paulo (SP): Fundação Getúlio Vargas; 2011.

e Dean AG SK, Soe MM. OpenEpi: Open Source Epidemiologic Statistics for Public Health. 2015 [cited 2015 Jun 22]. Available from: http://www.openepi.com/Menu/OE_Menu.htm

f Aguiar MESS. O impacto causado pela redução do IPI na arrecadação do ICMS no Brasil [dissertation]. Fortaleza (CE): Universidade Federal do Ceará; 2009.
